# Comparison between the Psychopathy Checklist-Revised and the Comprehensive Assessment of Psychopathic Personality in a representative sample of Spanish prison inmates

**DOI:** 10.1371/journal.pone.0228384

**Published:** 2020-02-05

**Authors:** Gerardo Flórez, Ventura Ferrer, Luis S. García, María R. Crespo, Manuel Pérez, Pilar A. Saiz, David J. Cooke

**Affiliations:** 1 Centro de Investigación Biomédica en Red de Salud Mental (CIBERSAM), Oviedo, Spain; 2 Health Department, Pereiro de Aguiar Prison, Ourense, Spain; 3 Department of Psychiatry, University of Oviedo, Oviedo, Spain; 4 Instituto de Investigación Sanitaria del Principado de Asturias (ISPA), Oviedo, Spain; 5 Mental Health Services of Principado de Asturias (SESPA), Oviedo, Spain; 6 Department of Psychosocial Science, University of Bergen, Bergen, Norway; Medical University of Vienna, AUSTRIA

## Abstract

In the field of psychopathy, there is an ongoing debate about the core traits that define the disorder, and that therefore must be present to some extent in all psychopaths. The main controversy of this debate concerns criminal behaviour, as some researchers consider it a defining trait, while others disagree. Using a representative sample of 204 Spanish convicted inmates incarcerated at the Pereiro de Aguiar Penitentiary in Ourense, Spain, we tested two competing models, the Psychopathy Checklist-Revised (PCL-R), which includes criminal behaviour items, versus the Comprehensive Assessment of Psychopathic Personality (CAPP), which does not. We used two different PCL-R models, one that includes criminal items and another that does not. PCL-R factors, facets, and testlets from both models and CAPP dimensions were correlated and compared. Two different PCL-R cut-off scores, 25 or more and 30 or more, were used for the analysis. Overall, a strong correlation was found between PCL-R and CAPP scores in the whole sample, but as scores increased and inmates became more psychopathic, the correlations weakened. All these data indicate that psychopathy, understood to mean having high scores on the PCL-R and CAPP, is a multidimensional entity, and inmates can develop the disorder and then receive the diagnosis through different dimensions. The CAPP domains showed better correlations when compared with the PCL-R factors from both models, showing that an instrument for the assessment of psychopathy without a criminal dimension is valuable for clinical assessment and research purposes.

## Introduction

Accurate diagnosis of Psychopathic Personality Disorder (PPD) or psychopathy is of great importance in clinical and forensic settings for risk management purposes [1, 2]. Previous research has established that psychopathy is one of the single strongest risk factors for violent behaviour and recidivism, and is the only mental disorder clearly related to instrumental violence [3–9].

But what exactly is psychopathy? Based on decades of research, it has been described as a severe form of personality disorder defined by a range of traits that include the following: (*affective*) callous—unemotional (CU) traits reflecting deficient affective experience, (*interpersonal*) grandiose and arrogant interpersonal style, and (*behavioural*) pervasive impulsive behaviour [5, 10–12]. What is the relationship among these traits in PPD? Is it categorical or dimensional, and which traits must be detected to confirm the diagnosis? [13, 14] These are the questions that clinical and forensic practitioners want researchers to answer unequivocally. In order to manage risk at the individual level, they need diagnostic precision and predictive utility [15–17].

In his highly influential book, Cleckey emphasized emotional and interpersonal traits that would lead to a series of antisocial, but not necessarily criminal behaviours. Today, many scholars still favour this point of view which does not consider criminality as core trait of psychopathy [3, 10, 18–22].

Other scholars also include criminal behaviour as a defining trait. Internationally considered the “state of the art” tool for the measurement of psychopathy, the *Psychopathy Checklist-Revised* (PCL-R) is exemplary of this point of view [5, 11]. It is a 20-item symptom-construct expert rating scale designed for use in forensic settings, with 3-point scale ratings for the lifetime presence and severity of each item. Data are collected through a semi-structured interview and review of file and collateral information. It also has a screening version that can be used in both civil and forensic populations. The author favours a 4-factor structure model: Factor 1 (*interpersonal/affective)* subdivided into Facet 1 (*interpersonal*) and Facet 2 (*affective*), and Factor 2 (*social deviance*) subdivided into Facet 3 (*lifestyle*) and Facet 4 (a*ntisocial) [23, 24]*. This model excludes two items. Good fit has been reported for the 4-factor structure model [11, 25, 26], but also for a 3-factor model (*arrogant and deceitful interpersonal style*, *deficient affective experience*, *and impulsive and irresponsible behavioural style*) proposed by other researchers before the author added the 4-facet structure [10, 25]. In this 3-factor model, which excludes seven items because they had weak overall associations with the latent trait, every factor has two “testlets”, single items or a combination of two items. Testlets were included in the model because item response theory analyses demonstrated that certain PCL-R items were more highly correlated than could be explained by their association with the underlying trait [10]. What is more important is that PCL-R items that measure criminality are excluded from the 3-factor model but included in the 4-factor one. Therefore, the PCL-R structural debate is also a debate about criminality as a core trait of psychopathy.

Authors who favour the idea that criminal behaviour should not be included as a defining trait point out that there is a clear bias in using it when defining PPD and afterwards using PPD to explain criminal behaviour, and that this overemphasis on criminality has obscured the nature of PPD as a personality disorder characterized by interpersonal aggression where criminal activity is not necessarily present [17, 20, 27, 28]. Some of these authors developed the *Comprehensive Assessment of Psychopathic Personality* (CAPP), a 33-item symptom-construct expert rating scale designed for use in forensic settings. This tool is organized into 6 domains (*Attachment*, *Behavioural*, *Cognitive*, *Dominance*, *Emotional*, and *Self*) [29, 30]. Each item is given a 7-point scale rating. The assessment timescale usually ranges from 6 to 12 months. Data are collected through a semi-structured interview, complemented by a file review and collateral information. Research has shown that CAPP validity is high across different cultures and languages [31–35]. One self-rating study reported best fit for a model with one general factor representing *global psychopathy*, and three residual factors: *boldness/emotional stability*, *emotional detachment*, and *disinhibition* [35]. Research has also shown that, using total and domain scores, the CAPP´s predictive validity for violent and nonviolent recidivism equals that of the PCL screening version [7].

Previous research comparing PCL-R and CAPP scores has shown a high correlation at the domain level with PCL-R total score [36], as would be expected.

Comparing PCL-R and CAPP scores in a large international non-convenience sample may help elucidate the previously mentioned debate concerning criminal behaviour as a core trait of psychopathy. The current study continues our previously published work in which PCL-R and CAPP scores were analysed independently [37, 38].

### Objectives

The current investigation was designed to compare PCL-R and CAPP scores in a sample of inmates incarcerated at the Pereiro de Aguiar Prison in Spain to test if they assess the same underlying psychopathy construct. Previous research points in this direction but convenience and small samples were used [36, 39]. The main hypothesis was that the CAPP, without considering criminal behaviour, would have a strong association with the PCL-R, suggesting that both tools are useful for assessing psychopathy.

## Materials and methods

### Participants and procedure

The protocol followed in the current study has been described in detail elsewhere [37, 38]. Participants in the current study are the same as in our previously published PCL-R and CAPP work [37, 38, 40].

As previously indicated the protocol was approved by the Pontevedra-Vigo-Ourense Local Research Ethics Committee (2014/009) [37, 38, 40]. Every participant provided written informed consent. The study was conducted in accordance with the Declaration of Helsinki. No financial or other compensation was offered. Participants in the study were able to opt out whenever they wanted to do so. As there was no research treatment in the study, all inmates, whether participants or not, received the same treatments.

Table 1 provides a summary of the IPDE (International Personality Disorder Examination) scores and sociodemographic and forensic variables of the sample. The PCL-R European cut-off score of 25 is used. The CAPP was not designed to have a cut-off, as its purpose is to provide a comprehensive clinical formulation, but, in Table 1, and for comparison purposes in the current study, a cut-off score of 124 was used for the CAPP. It was chosen because, within the CAPP, it is the same percentile as the PCL-R cut-off score of 25.

**Table 1:** As previously reported in [37, 38, 40], Table 1 indicates IPDE scores and sociodemographic and forensic variables of the sample. Significance levels of these variables in relation to a PCL-R cut-off score of 25 and a proportional CAPP cut-off score of 124 are also shown.

Variables	% of inmates	PCL-R of 25 or more	CAPP of 124 or more
**Sex**			
**Male**	176 (86.27%)	69 (24.41%)	44 (21.57%)
**Female**	28 (13.73%)	11 (4.43%) X^2^ = 0.0001 p = 0.99	3 (1.47%) p = 0.14
**Age (mean (SD))**	40.93 (11.18)	39.98 (10.69)	46.96 (8.80)
		T = -1.02 p = 0.30	T = 4.98 p<0.001
**Nationality**			
**Spanish**	179 (87.75)	68 (33.33%)	44 (21.57%)
**Others**	25 (12.25)	12 (5.88%) X^2^ = 0.09 p = 0.33	3 (1.47%) p = 0.20
**Education years completed (mean, (SD))**			
**Basic**	8.84 (1.95)	8.77 (1.93) T = -0.28 p = 0.77	8.04 (1.82) T = -3.30 p = 0.001
**Higher**	0.24 (0.88)	0.10 (0.30) T = 0.67 p = 0.50	0.11 (0.52) T = -1.58 p = 0.11
**Marital status**			
**Married**	49 (24.01)	7 (2.45%)	6 (2.94%)
**Separated/divorced**	61 (29.9)	26 (12.75%)	21 (10.29%)
**Widowed**	1 (0.51)	0 (0%)	1 (0.49%)
**Single**	93 (45.58)	47 (18.63%) X^2^ = 18.71 p = 0.0003	19 (9.31%) p = 0.008
**Total months in prison (mean (SD))**	75.08 (83.56)	106.69 (86.33) T = -4.41 p<0.001	201.57 (80.84) T = 13.66 p<0.001
**Drug /Alcohol use**			
**Alcohol**	165 (80.88)	65 (31.86%) X^2^ = 0.01 p = 0.91	43 (21.08%) p = 0.0825
**Alcohol abuse**	78 (38.24)	25 (12.25%) X2 = 0.03 p = 0.91	16 (7.84%) X^2^ = 63.44 p = 0.001
**Heroin**	90 (44.12)	53 (25.98%) X^2^ = 18.06 p = 0.02	28 (13.73%) X^2^ = 0.07 p = 0.858
**Methadone**	70 (34.31)	43 (21.08%) X^2^ = 20.60 p = 0.02	24 (11.76%) X^2^ = 7.60 p = 0.01
**Other Opiates**	15 (7.35)	11 (5.39%) X^2^ = 7.90 p = 0.009	4 (1.96%) p = 0.858
**Benzodiacepines**	38 (18.63)	24 (11.76%) X^2^ = 11.23 p = 0.002	12 (5.98%) X^2^ = 1.92 p = 0.26
**Cocaine**	125 (61.27)	60 (29.41%) X^2^ = 10.44 p = 0.002	29 (14.22%) X^2^ = 0.005 p = 0.94
**Amphetamines**	28 (13.73)	16 (7.84%) X^2^ = 4.37 p = 0.04	10 (4.90%) X^2^ = 2.94 p = 0.17
**Cannabis**	117 (57.35)	59 (28.92%) X^2^ = 14.46 p = 0.002	31 (15.20%) X^2^ = 1.84 p = 0.26
**Hallucinogens**	30 (14.71)	18 (8.82%) X^2^ = 6.37 p = 0.01	9 (4.41%) X^2^ = 8.09 p = 0.01
**Inhalants**	7 (3.43)	4 (1.96%) X^2^ = 0.97 p = 0.39	3 (1.47%) p = 0.27
**Two or more**	142 (60.61)	68 (33.33%) X^2^ = 14.73 p<0.001	39 (19.12%) X^2^ = 5.16 p = 0.02
**Three or more**	112 (54.90)	59 (28.92%) X^2^ = 18.88 p<0.001	34 (16.66%) X^2^ = 7.50 p = 0.006
**Four or more**	92 (45.10)	51 (25.00%) X^2^ = 18.49 p<0.001	29 (14.22%) X^2^ = 6.80 p = 0.009
**Two or more (neither alcohol nor methadone)**	114 (55.88)	60 (29.41%) X^2^ = 19.51 p<0.001	30 (14.71%) X^2^ = 1.56 p = 0.21
**Three or more (neither alcohol nor methadone)**	86 (42.16)	46 (22.55%) X^2^ = 12.70 p<0.001	26 (12.75%) X^2^ = 4.33 p = 0.03
**Four or more (neither alcohol nor methadone)**	49 (24.02)	33 (16.18%) X^2^ = 21.41 p<0.001	16 (7.84%) X^2^ = 3.36 p = 0.06
**Type of official charges**			
**Drug dealing**	79 (38.73)	44 (21.57%) X^2^ = 14.69 p = 0.003	26 (12.75%) X^2^ = 7.08 p = 0.23
**Crimes against property**	116 (56.86)	61 (29.90%) X^2^ = 20.16 p = 0.003	35 (17.16%) X^2^ = 7.71 p = 0.05
**Violent crimes**	91 (44.61)	44 (21.57%) X^2^ = 5.75 p = 0.01	21 (10.29%) X^2^ = 0.001 p = 0.99
**Other crimes**	54 (26.47)	22 (10.78%) X^2^ = 0.07 p = 0.86	7 (3.43%) X^2^ = 4.20 p = 0.20
**Disorderly conduct**	32 (15.69)	13 (6.37%) X^2^ = 0.03 p = 0.86	4 (1.96%) p = 0.88
**Driving while intoxicated**	42 (20.59)	8 (3.92%) X^2^ = 9.02 p = 0.006	6 (2.94%) X^2^ = 2.28 p = 0.32
**Major driving violations**	60 (29.41)	23 (11.27%) X^2^ = 0.02 p = 0.83	13 (6.37%) X^2^ = 0.09 p = 0.88
**Two or more**	149 (73.04)	71 (34.80%) X^2^ = 16.49 p = 0.003	35 (17.16%) X^2^ = 0.06 p = 0.88
**Three or more**	81 (39.71)	42 (20.59%) X^2^ = 8.99 p = 0.006	20 (9.80%) X^2^ = 0.20 p = 0.88
**Four or more**	26 (12.75)	16 (7.84%) X^2^ = 6.22 p = 0.01	7 (3.43%) X^2^ = 0.25 p = 0.88
**IPDE diagnosis**			
**Paranoid**	29 (14.22)	15 (7.35%) X^2^ = 2.21 p = 0.43	15 (7.35%) X^2^ = 19.68 p = 0.01
**Schizoid**	0 (0)	0 (0%) p = 1	0 (0%) p = 1
**Schizotypal**	1 (0.49)	1 (0.49%) p = 0.72	1 (0.49%) p = 0.82
**Antisocial**	38 (18.63)	30 (14.71%) X^2^ = 30.92 p = 0.005	17 (8.33%) X^2^ = 12.39 p = 0.002
**Borderline**	15 (7.35)	8 (3.92%) X^2^ = 1.35 p = 0.6	6 (2.94%) X^2^ = 0.44 p = 0.83
**Histrionic**	13 (6.37)	6 (2.94%) X^2^ = 0.28 p = 0.73	4 (1.96%) p = 0.83
**Narcissistic**	43 (21.08)	29 (14.22%) X^2^ = 18.21 p = 0.005	25 (12.25%) X^2^ = 37.85 p = 0.003
**Avoidant**	17 (8.33)	5 (2.45%) p = 0.72	3 (1.47%) p = 0.99
**Dependent**	2 (0.98)	0 (0%) p = 0.51	0 (0%) p = 1
**Obsessive**	2 (0.98)	1 (0.49%) p = 1	0 (0%) p = 1
**More than one**	103 (50.49)	58 (28.43%) X^2^ = 25.50 p<0.001	27 (13.24%) X^2^ = 1.18 p = 0.27

PCL-R: Psychopathy Checklist-Revised; CAPP: Comprehensive Assessment of Psychopathic Personality; SD: Standard Deviation

### Analyses

R software, version 3.4.3, was used for all analyses (https://www.r-project.org/)[41]. Means and standard deviations and percentages were calculated for continuous and categorical variables, respectively. Group differences were found using two-sample t (Welch’s t-test), chi-square and ANOVA tests.

Linear regression analyses were applied using total PCL-R and total CAPP. All variables from Table 1 were used in the models. This analysis was done for the whole sample only. Sub-samples above the PCL-R cut-offs were too small.

We used a series of regressions with the step wise and Ridge methods to predict PCL-R factors, facets, and testlets from CAPP domains and vice versa. This analysis was done for the whole sample only. Sub-samples above the PCL-R cut-offs were too small.

The false discovery rate is controlled with the help of the method proposed by Benjamini and Hochberg. It consists of a simple sequential Bonferroni type procedure that can control the false discovery rate for independent test statistics. All the p-values obtained in the contrast hypothesis tests performed in this study have been corrected using this method.

## Results

Table 1 indicates that PCL-R scores do not seem to be influenced by sex, age, nationality, or education; but do seem to be influenced by marital status (lower for married people and higher for singles), total time in prison (higher for longer stays), use of alcohol/drugs (in general, higher for more use), type of official charges (drug dealing, property crimes, violent crimes and more than one type were positively associated with PCL-R scores, and driving while intoxicated correlated negatively), and IPDE diagnosis (Antisocial, Narcissistic, and more than one were positively associated with PCL-R scores). Table 1 also shows that CAPP scores do not seem to be influenced by sex or nationality, but do seem to be influenced by age (higher for older age), education (higher for fewer years), marital status (same results as PCL-R), total stay in prison (same as PCL-R), use of alcohol/drugs (alcohol abuse, methadone, hallucinogens, and more than one substance were positively associated with CAPP scores), type of official charges (property crimes were positively associated), and IPDE diagnosis (Paranoid, Antisocial, and Narcissistic were positively associated).

Using a PCL-R European standard cut-off score of 25 or more (Cooke, 1995; Cooke, Hart, & Michie, 2004), 80 (39.21%) inmates met the criteria for psychopathy, and 28 (13.73%) met those criteria when using the North American/Canadian standard cut-off score of 30 or more. Thus, 42 (20.58%) of the inmates had a CAPP total score greater than or equal to 124 (of these, 34 (80.95%) had a PCL-R total score greater than or equal to 25); and 162 (79.42%) had a score lower than 124 (of these, 46 (37.09%) had a PCL-R total score greater than or equal to 25) (p<0.001). When using a CAPP cut-off score of 148.5, proportional to the PCL-R cut-off of30, 11 (5.39%) were below the cut-off score (of these, 7 (63.63%) had a PCL-R total score greater than or equal to 30); and 193 (94.61%) had a score a lower than 148.5 (of these, 20 (10.88%) had a PCL-R total score greater than or equal to 30) (p<0.001).

For the PCL-R regression analysis model, in which all variables from Table 1 and CAPP scores were used (total and item scores), the following variables were significant at an alpha level of 5% for the whole sample: CAPP Total, mean age of first alcohol use, drug dealing, property crimes, violent crimes, driving while intoxicated, major driving violations and IPDE Avoidant.

For the CAPP regression analysis, in which all variables from Table 1 and PCL-R scores were used (total and item scores), the following variables were significant at an alpha level of 5% for the whole sample: PCL-R Total, IPDE Paranoid, IPDE Antisocial, IPDE Borderline and IPDE Narcissistic.

Table 2 shows correlations between PCL-R (Total, Factor, and Facet scores following the Hare model) and CAPP (Total and Domain scores) for the whole sample, for inmates with a PCL-R of 25 or more and for inmates with a PCL-R of 30 or more.

**Table 2:** As previously reported in [38], Table 2 indicates correlations between PCL-R (total, factor and facet scores following the Hare model) and CAPP (total and domain scores) for the whole sample, for inmates with a European PCL-R cut-off score of 25 or more (italics), and for inmates with a North American/Canadian PCL-R cut-off score of 30 or more (underlined).

	**PCL Total**	**PCL Factor1**	**PCL Factor2**	**PCL Facet1**	**PCL Facet2**	**PCL Facet3**	**PCL Facet4**
**PCL Factor1**	0.83**	**1.00**					
	*0*.40**						
	0.54*						
**PCL Factor2**	0.86**	0.46**	**1.00**				
	*0*.65**	*-0*.28*					
	0.66**	-0.03					
**PCL Facet1**	0.75**	0.89**	0.43**	**1.00**			
	*0*.34*	*0*.79**	*-0*.26*				
	0.30	0.66**	-0.17				
**PCL Facet2**	0.72**	0.88**	0.39**	0,.59**	**1.00**		
	*0*.24*	*0*.66**	*-0*.14*	*0*.*08*			
	0.40	0.62**	0.12	-0.12			
**PCL Facet3**	0.82**	0.46**	0.92**	0.46**	0.36**	**1.00**	
	*0*.39**	*-0*.31*	*0*.67**	*-0*.*21*	*-0*.25*		
	0.35	0.07	0.27	0.1	0.02		
**PCL Facet4**	0.72**	0.35**	0.88**	0.29**	0.34**	0.64**	**1.00**
	*0*.61**	*-0*.*18*	*0*.89**	*-0*.*21*	*-0*.*03*	*0*.28*	
	0.60*	-0.05	0.97**	-0.20	0.12	0.06	
**CAPP Attachment**	0.59**	0.63**	0.40**	0.44**	0.69**	0.32**	0.42**
	*0*.*17*	*0*.*18*	*0*.*11*	*-0*.*17*	*0*.51**	*-0*.*14*	*0*.23*
	0.46*	0.21	0.46*	-0.23	0.53*	0.31	0.41*
**CAPP Behavioural**	0.61**	0.41**	0.64**	0.37**	0.36**	0.59**	0.57**
	*0*.24*	*-0*.*20*	*0*.44**	*-0*.*15*	*-0*.*15*	*0*.26*	*0*.41**
	0.54*	0.15	0.46*	0,07	0.13	0.38	0.40*
**CAPP Cognitive**	0.58**	0.48**	0.51**	0.37**	0.49**	0.46**	0.47**
	*0*.*10*	*-0*.24*	*0*.30*	*-0*.34*	*0*.*02*	*0*.*10*	*0*.33*
	0.29	-0.01	0.3*5	-0.31	0.18	0.30	0.30*
**CAPP Dominance**	0.67**	0.72**	0.45**	0.61**	0.67**	0.40**	0.42**
	*0*.27*	*0*.25*	*0*.*07*	*0*.*06*	*0*.34*	*-0*.*20*	*0*.21*
	0.38*	0,05	0.34	-0.09	0.19	0.22	0.31
**CAPP Emotional**	0.63**	0.60**	0.49**	0.43**	0.65**	0.43**	0.47**
	*0*.*17*	*0*.*03*	*0*.*18*	*-0*.27*	*0*.39**	*-0*.*07*	*0*.29*
	0.37*	0.08	0.38*	-0.28	0.42*	0.18	0.33*
**CAPP Self**	0.61**	0.66**	0.40**	0.63**	0.55**	0.36**	0.37**
	*0*.38**	*0*.33*	*0*.*10*	*0*.32*	*0*.*16*	*-0*.*02*	*0*.*14*
	0.20	0.10	0.16	0.13	0.01	0.16	0.13
**CAPP Total**	0.71*	0.68*	0.55**	0.57*	0.65*	0.49**	0.51**
	*0*.30*	*0*.*10*	*0*.*24*	*-0*.*07*	*0*.26*	*-0*.*02*	*0*.33**
	0.44*	0.10	0.41*	-0.09	0.24	0.30	0.35*

PCL-R: Psychopathy Checklist-Revised; CAPP: Comprehensive Assessment of Psychopathic Personality;

* 0.05 < p ≥ 0.001;

** p<0.001

Table 3 shows correlations between PCL-R (Total, Factor and Testlet scores following the Cooke and Michie model) and CAPP (Total and Domain scores) for the whole sample, for inmates with a PCL-R of 25 or more and for inmates with a PCL-R of 30 or more.

**Table 3:** As previously indicated in [38], Table 3 indicates correlations between PCL-R (total, factor and testlet scores following the Cooke and Michie model) and CAPP (total and domain scores) for the whole sample, for inmates with a PCL-R cut-off score of 25 or more (italics), and for inmates with a PCL-R cut-off score of 30 or more (underlined).

	**PCL Total**	**PCL Testlet1**	**PCL Testlet2**	**PCL Testlet3**	**PCL Testlet4**	**PCL Testlet5**	**PCL Testlet6**	**PCL Factor1**	**PCL Factor2**	**PCL Factor3**
**PCL Testlet1**	0.61**	**1.00**								
	*0*.34*									
	0.02									
**PCL Testlet2**	0.69**	0.49**	**1.00**							
	*0*.*15*	*0*.*19*								
	0.47*	0.21								
**PCL Testlet3**	0.63**	0.42**	0.49**	**1.00**						
	*0*.25*	*0*.*05*	*0*.*09*							
	0.38*	-0.24	0.06							
**PCL Testlet4**	0.59**	0.37**	0.43**	0.43**	**1.00**					
	*-0*.*02*	*-0*.*14*	*0*.*13*	*-0*.*05*						
	0.18	-0.11	-0.11	0.19						
**PCL Testlet5**	0.66**	0.21**	0.32**	0.18**	0,19**	**1.00**				
	*0*.25*	*-0*.*12*	*-0*.*14*	*-0*.25*	*-0*.*05*					
	0.12	-0.20	-0.11	0.20	-0.04					
**PCL Testlet6**	0.76*	0.36**	0.50**	0.32**	0,37**	0.48**	**1.00**			
	*0*.32*	*-0*.*19*	*-0*.*01*	*-0*.*16*	*0*.*14*	*0*.*13*				
	0.33	0.10	0.14	-0.11	0.29	-0.06				
**PCL Factor1**	0.75**	0.86**	0.86**	0.53**	0,47**	0.31**	0.50**	**1.00**		
	*0*.34*	*0*.88**	*0*.62**	*0*.*08*	*-0*.*05*	*-0*.*18*	*-0*.*15*			
	0.32	0.80**	0.75**	-0.12	-0.15	-0.15	0.15			
**PCL Factor2**	0.72**	0.47**	0.55**	0.87**	0,80**	0.22**	0.41**	0.59**	**1.00**	
	*0*.24*	*0*.*03*	*0*.*11*	*0*.98**	*0*.*10*	*-0*.27*	*-0*.*13*	*0*.*08*		
	0.40*	-0.24	0.05	0.98**	0.33	0.17	-0.06	-0.13		
**PCL Factor3**	0.82**	0.33**	0.48**	0.29**	0,32**	0.87**	0.85**	0.46**	0.36**	**1.00**
	*0*.39**	*-0*.22*	*-0*.*08*	*-0*.26*	*0*.*08*	*0*.63**	*0*.85**	*-0*.21*	*-0*.25*	
	0.35	0.05	0.10	-0.03	0.27	0.26	0.93**	0.1	0.003	
**CAPP Attachment**	0.59**	0.35**	0.42**	0.69**	0,46**	0.22**	0.34**	0.44**	0.69**	0.32**
	*0*.*17*	*-0*.*12*	*0*.*15*	*0*.52**	*-0*.*01*	*-0*.24*	*-0*.*02*	*-0*.*17*	*0*.51**	*-0*.*14*
	0.46*	-0.37	0.01	0.49*	0.42*	0.12	0.27	-0.23	0.54*	0.30
**CAPP Behavioural**	0.61**	0.28**	0.36**	0.29**	0,32**	0.48**	0.55**	0.37**	0.36**	0.59**
	*0*.24*	*-0*.*14*	*-0*.*08*	*-0*.*15*	*0*.*03*	*0*.*20*	*0*.*19*	*-0*.*15*	*-0*.*15*	*0*.26*
	0.54*	-0.15	0.35	0.08	0.43*	0.14	0.35	0.07	0.13	0.38*
**CAPP Cognitive**	0.58**	0.29**	0.34**	0.41**	0,43**	0.28**	0.51**	0.37**	0.49**	0.46**
	*0*.*10*	*-0*.28*	*-0*.24*	*0*.*02*	*-0*.*02*	*-0*.*09*	*0*.*19*	*-0*.34*	*0*.*02*	*0*.*10*
	0.30	-0.50*	0.02	0.13	0.41*	0.21	0.24	-0.32	0.20	0.31
**CAPP Dominance**	0.67**	0.49**	0.57**	0.63**	0,49**	0.26**	0.45**	0.61**	0.67*	0.40**
	*0*.27*	*0*.*06*	*0*.*03*	*0*.33*	*0*.*05*	*-0*.24*	*-0*.*08*	*0*.*06*	*0*.34*	*-0*.20*
	0.37*	-0.31	0.16	0.11	0.54*	0.01	0.23	-0.11	0.18	0.22
**CAPP Emotional**	0.63**	0.34**	0.40**	0.62**	0.46**	0.30**	0.45**	0.43**	0.65**	0.43**
	*0*.*17*	*-0*.*21*	*-0*.22*	*0*.39**	*0*.*01*	*-0*.*13*	*-0*.*01*	*-0*.27*	*0*.39**	*-0*.*07*
	0.37*	-0.46*	0.04	0.36*	0.51*	0.05	0.18	-0.29	0.43*	0.19
**CAPP Self**	0.61**	0.60**	0.49**	0.48**	0,44**	0.25**	0.38**	0.63**	0.55**	0.36**
	*0*.38**	*0*.34*	*0*.*10*	*0*.*15*	*0*.*06*	*-0*.*13*	*0*.*06*	*0*.32*	*0*.*16*	*-0*.*02*
	0.20	0.002	0.23	-0.06	0.40*	-0.07	0.20	0.14	0.01	0.17
**CAPP Total**	0.71**	0.47**	0.51**	0.59**	0,50**	0.34**	0.51**	0.57**	0.65**	0.49**
	*0*.30*	*-0*.*03*	*-0*.*08*	*0*.25*	*0*.*03*	*-0*.*13*	*0*.*05*	*-0*.*07*	*0*.26*	*-0*.*02*
	0.44*	-0.31	0.17	0.17	0.53*	0.08	0.29	-0.10	0.24	0.30

PCL-R: Psychopathy Checklist-Revised; CAPP: Comprehensive Assessment of Psychopathic Personality;

* p <0.05;

** p≤ 0.001

The Attachment and the Emotional domains showed their best correlations with interpersonal/affective (Factor 1, Hare model) and affective (Facet 2, Hare model) and also with deficient affective experience (Factor 2, Cooke and Michie model) and Testlet 3 (items 7 and 8). The Dominance domain showed its best correlations with interpersonal/affective (Factor 1, Hare model) interpersonal (Facet 1, Hare model) and affective (Facet 2, Hare model), and also, with arrogant and deceitful interpersonal style (Factor 1, Cooke and Michie model) deficient affective experience (Factor 2, Cooke and Michie model) and Testlet 3. The Cognitive domain retains its correlations in a neutral position between factors, facets and testlets. The Self domain showed its best correlations with interpersonal/affective (Factor 1, Hare model) interpersonal (Facet 1, Hare model) and affective (Facet 2, Hare model), and also, with arrogant and deceitful interpersonal style (Factor 1, Cooke and Michie model) and Testlet 1 (items 1 and 2). The Behavioural domain showed its best correlations with social deviance (Factor 2, Hare model) and lifestyle (Facet 3) and antisocial (Facet 4), and also, with impulsive and irresponsible behavioural style (Factor 3, Cooke and Michie model) and Testlet 6 (items 9 and 13).

When the PCL-R cut-off score of 25 was used, these correlations were found to be relevant: the Attachment and Emotional domains with affective (Facet 2, Hare model) and also with deficient affective experience (Factor 2, Cooke and Michie model) and Testlet 3; the Dominance domain showed its best correlations with interpersonal/affective (Factor 1, Hare model) and affective (Facet 2, Hare model); and also with arrogant and deceitful interpersonal style (Factor 1, Cooke and Michie model), deficient affective experience (Factor 2, Cooke and Michie model) and Testlet 3; the Self domain showed its best correlations with interpersonal / affective (Factor 1, Hare model), interpersonal (Facet 1, Hare model), and also with arrogant and deceitful interpersonal style (Factor 1, Cooke and Michie model), and Testlet 1; the Behavioural domain showed its best correlations with social deviance (Factor 2, Hare model) and antisocial (Facet 4), and also with impulsive and irresponsible behavioural style (Factor 3, Cooke and Michie model).

When the PCL-R cut-off score of 30 was used, these correlations were found to be relevant: Attachment and Emotional domains with affective (Facet 2, Hare model) and also with deficient affective experience (Factor 2, Cooke and Michie model) and Testlets 3 and 4; the Dominance domain and Testlet 4; the Cognitive domain and Testlet 4; the Self domain and Testlet 4; while the behavioural domain showed its best correlations with social deviance (Factor 2, Hare model) and antisocial (Facet 4), and also with impulsive and irresponsible behavioral style (Factor 3, Cooke and Michie model) and Testlet 4.

In order to more precisely compare the higher-level dimensions measured by the two psychopathy assessment tools, we used a series of linear regressions with the step-wise method to predict PCL-R factors, facets, and testlets from the CAPP domains, and vice versa (Tables 4 and 5).

**Table 4:** Regressions with the stepwise method predicting each higher-level dimension of both instruments (PCL-R, Hare model).

CAPP	B	P	PCL-R	B	P
**Attachment**			**Factor 1**		
**Factor 1**	0.57	p≤ 0.001	**Attachment**	0.29	p≤ 0.001
**Factor 2**	0.14	p≤ 0.001	**Cognitive**	-0.18	0.01
			**Dominance**	0.48	p≤ 0.001
			**Self**	0.18	0.02
*R*^*2*^	0.42		*R*^*2*^	0.57	
**Behavioural**			**Factor 2**		
**Factor 1**	0.14	p≤ 0.001	**Behavioural**	0.56	p≤ 0.001
**Factor 2**	0.58	p≤ 0.001	**Emotional**	0.25	0.002
R^2^	0.43		*R*^*2*^	0.44	
**Cognitive**			**Facet 1**		
**Factor 1**	0.31	p≤ 0.001	**Cognitive**	-0.17	0.02
**Factor 2**	0.36	p≤ 0.001	**Dominance**	0.62	p≤ 0.001
			**Self**	0.34	p≤ 0.001
*R*^*2*^	0.34		*R*^*2*^	0.52	
**Dominance**			**Facet 2**		
**Factor 1**	0.70	p≤ 0.001	**Attachment**	0.46	p≤ 0.001
**Factor 2**	0.04	p≤ 0.001	**Cognitive**	-0.18	0.02
			**Dominance**	0.20	p≤ 0.001
			**Emotional**	0.27	0.004
*R*^*2*^	0.52		*R*^*2*^	0.53	
**Emotional**			**Facet 3**		
**Factor 1**	0.47	p≤ 0.001	**Behavioural**	0.54	p≤ 0.001
**Factor 2**	0.27	p≤ 0.001	**Emotional**	0.21	0.02
*R*^*2*^	0.42		*R*^*2*^	0.37	
**Self**			**Facet 4**		
**Factor 1**	0.60	p≤ 0.001	**Behavioural**	0.48	p≤ 0.001
**Factor 2**	0.1244	p≤ 0.001	**Dominance**	-0.16	p≤ 0.001
			**Emotional**	0.30	p≤ 0.001
*R*^*2*^	0.45		*R*^*2*^	0.36	
**Attachment**					
**Facet 2**	0.62	p≤ 0.001			
**Facet 4**	0.21	p≤ 0.001			
*R*^*2*^	0.52				
**Behavioural**					
**Facet 2**	0.12	p≤ 0.001			
**Facet 3**	0.35	p≤ 0.001			
**Facet 4**	0.29	p≤ 0.001			
*R*^*2*^	0.43				
**Cognitive**					
**Facet 2**	0.34	p≤ 0.001			
**Facet 3**	0.18	0.01			
**Facet 4**	0.23	p≤ 0.001			
*R*^*2*^	0.36				
**Dominance**					
**Facet 1**	0.52	p≤ 0.001			
**Facet 2**	0.28	p≤ 0.001			
**Facet 3**	-0.10	0.13			
**Facet 4**	0.14	p≤ 0.001			
*R*^*2*^	0.54				
**Emotional**					
**Facet 2**	0.55	p≤ 0.001			
**Facet 4**	0.28	p≤ 0.001			
*R*^*2*^	0.49				
**Self**					
**Facet 1**	0.45	p≤ 0.001			
**Facet 2**	0.22	p≤ 0.001			
**Facet 4**	0.15	p≤ 0.001			
*R*^*2*^	0.47				

PCL-R: Psychopathy Checklist-Revised; CAPP: Comprehensive Assessment of Psychopathic Personality

**Table 5:** Regressions with the stepwise method predicting each higher-level dimension of both instruments (PCL-R, Cooke and Michie model).

CAPP	B	P	PCL-R	B	P
**Attachment**			**Factor 1**		
**Factor 2**	0.66	p≤ 0.001	**Cognitive**	-0.17	0.02
			**Dominance**	0.62	p≤ 0.001
			**Self**	0.34	p≤ 0.001
*R*^*2*^	0.49		*R*^*2*^	0.52	
**Behavioural**			**Factor 2**		
**Factor 2**	0.16	0.005	**Attachment**	0.46	p≤ 0.001
**Factor 3**	0.53	p≤ 0.001	**Cognitive**	-0.18	0.02
			**Dominance**	0.20	p≤ 0.001
			**Emotional**	0.27	p≤ 0.001
*R*^*2*^	0.38		*R*^*2*^	0.53	
**Cognitive**			**Factor 3**		
**Factor 2**	0.37	p≤ 0.001	**Behavioural**	0.54	p≤ 0.001
**Factor 3**	0.32	p≤ 0.001	**Emotional**	0.21	0.02
*R*^*2*^	0.33		*R*^*2*^	0.37	
**Dominance**			**Testlet 1**		
**Factor 1**	0.50	p≤ 0.001	**Cognitive**	-0.17	0.05
**Factor 2**	0.30	p≤ 0.001	**Dominance**	0.33	p≤ 0.001
			**Emotional**	-0.20	0.03
			**Self**	0.60	p≤ 0.001
*R*^*2*^	0.53		*R*^*2*^	0.43	
**Emotional**			**Testlet 2**		
**Factor 2**	0.56	p≤ 0.001	**Behavioural**	0.14	p≤ 0.001
**Factor 3**	0.22	p≤ 0.001	**Cognitive**	-0.25	0.007
			**Dominance**	0.72	p≤ 0.001
*R*^*2*^	0.46		*R*^*2*^	0.41	
**Self**			**Testlet 3**		
**Factor 1**	0.47	p≤ 0.001	**Attachment**	0.57	p≤ 0.001
**Factor 2**	0.26	p≤ 0.001	**Cognitive**	-0.34	p≤ 0.001
			**Dominance**	0.24	p≤ 0.001
			**Emotional**	0.36	p≤ 0.001
*R*^*2*^	0.45		*R*^*2*^	0.55	
**Attachment**			**Testlet 4**		
**Testlet 3**	0.59	p≤ 0.001	**Attachment**	0.28	0.001
**Testlet 4**	0.17	p≤ 0.001	**Dominance**	0.25	p≤ 0.001
*R*^*2*^	0.52		*R*^*2*^	0.24	
**Behavioural**			**Testlet 5**		
**Testlet 4**	0.13	0.02	**Behavioural**	0.60	p≤ 0.001
**Testlet 5**	0.27	p≤ 0.001	**Cognitive**	-0.24	0.03
**Testlet 6**	0.36	p≤ 0.001	**Emotional**	0.22	0.04
*R*^*2*^	0.37		*R*^*2*^	0.26	
**Cognitive**			**Testlet 6**		
**Testlet 3**	0.20	0.001	**Behavioural**	0.37	p≤ 0.001
**Testlet 4**	0.20	0.001	**Cognitive**	0.19	0.08
**Testlet 6**	0.37	p≤ 0.001	**Emotional**	0.19	0.07
*R*^*2*^	0.36		*R*^*2*^	0.34	
**Dominance**					
**Testlet 1**	0.24	p≤ 0.001			
**Testlet 2**	0.34	p≤ 0.001			
**Testlet 3**	0.24	p≤ 0.001			
**Testlet 4**	0.11	p≤ 0.001			
*R*^*2*^	0.53				
**Emotional**					
**Testlet 3**	0.47	p≤ 0.001			
**Testlet 4**	0.16	p≤ 0.001			
**Testlet 6**	0.19	p≤ 0.001			
*R*^*2*^	0.48				
**Self**					
**Testlet 1**	0.40	p≤ 0.001			
**Testlet 2**	0.13	p≤ 0.001			
**Testlet 3**	0.17	0.006			
**Testlet 4**	0.16	0.007			
*R*^*2*^	0.46				

PCL-R: Psychopathy Checklist-Revised; CAPP: Comprehensive Assessment of Psychopathic Personality

That analysis showed the following clear associations for the Hare model: Attachment and Factor 1 & Facet 2; Behavioural and Factor 2; Dominance and Factor 1 & Facet 1 & Facet 2; Emotional and Facet 2; and, Self and Factor 1 & Facet 1; and the following for the Cooke and Michie model: Attachment and Factor 2 & Testlet 3; Dominance and Factor 1 & Factor 2 & Testlet 1 & Testlet 2 & Testlet 3; Emotional and Factor 2 & Testlet 3; and Self and Factor 1 & Testlet 1. The following CAPP domains were not predicted with an *R*^*2*^ above 0.40 (Tables 4 and 5): Cognitive with Hare´s facets, and Behavioural and Cognitive with Cooke and Michie´s factors and testlets. The following PCL-R factors, facets and testlets were not predicted with an *R*^*2*^ above 0.40 (Tables 4 and 5): Hare´s Facet 3 and Facet 4, and Cooke and Michie´s Factor 3, Testlet 4, Testlet 5, and Testlet 6. Regressions with the Enter method provided very similar *R*^2^ values.

To verify the linear regressions with the step-wise method results we performed a Ridge regression (Tables 6 and 7).

**Table 6:** Ridge regression predicting each higher-level dimension of both instruments (PCL-R, Hare model).

CAPP	B	P	PCL-R	B	P
**Attachment**			**Factor 1**		
**Factor 1**	0.70	p≤ 0.001	**Attachment**	0.20	p≤ 0.001
**Factor 2**	0.16	p≤ 0.001	**Cognitive**	-0.13	0.03
			**Dominance**	0.22	p≤ 0.001
			**Self**	0.10	0.03
*R*^*2*^	0.42		*R*^*2*^	0.57	
**Behavioural**			**Factor 2**		
**Factor 1**	0.25	0.013	**Behavioural**	0.33	p≤ 0.001
**Factor 2**	0.92	p≤ 0.001	**Emotional**	0.24	0.005
*R^2^*	0.43		*R*^*2*^	0.44	
**Cognitive**			**Facet 1**		
**Factor 1**	0.38	p≤ 0.001	**Cognitive**	-0.07	0.02
**Factor 2**	0.41	p≤ 0.001	**Dominance**	0.12	p≤ 0.001
			**Emotional**	-0.14	p≤ 0.001
			**Self**	0.11	p≤ 0.001
*R*^*2*^	0.34		*R*^*2*^	0.50	
**Dominance**			**Facet 2**		
**Factor 1**	1.75	p≤ 0.001	**Attachment**	0.19	p≤ 0.001
**Factor 2**	0.39	p≤ 0.001	**Dominance**	0.016	p≤ 0.001
*R*^*2*^	0.53		*R*^*2*^	0.53	
**Emotional**			**Facet 3**		
**Factor 1**	0.59	p≤ 0.001	**Behavioural**	0.19	p≤ 0.001
**Factor 2**	0.32	p≤ 0.001	**Emotional**	0.11	0.03
*R*^*2*^	0.42		*R*^*2*^	0.37	
**Self**			**Facet 4**		
**Factor 1**	1.19	p≤ 0.001	**Behavioural**	0.12	p≤ 0.001
**Factor 2**	0.23	0.02	**Emotional**	0.10	0.005
*R*^*2*^	0.45		*R*^*2*^	0.36	
**Attachment**					
**Facet 2**	1.35	p≤ 0.001			
**Facet 4**	0.59	p≤ 0.001			
*R*^*2*^	0.53				
**Behavioural**					
**Facet 3**	0.84	p≤ 0.001			
**Facet 4**	1.01	p≤ 0.001			
*R*^*2*^	0.43				
**Cognitive**					
**Facet 2**	0.72	p≤ 0.001			
**Facet 3**	0.31	0.01			
**Facet 4**	0.55	p≤ 0.001			
*R*^*2*^	0.36				
**Dominance**					
**Facet 1**	1.50	p≤ 0.001			
**Facet 2**	2.05	p≤ 0.001			
**Facet 4**	1.00	p≤ 0.001			
*R*^*2*^	0.55				
**Emotional**					
**Facet 2**	1.20	p≤ 0.001			
**Facet 4**	0.56	p≤ 0.001			
*R*^*2*^	0.50				
**Self**					
**Facet 1**	1.58	p≤ 0.001			
**Facet 2**	0.82	p≤ 0.001			
**Facet 4**	0.68	p≤ 0.001			
*R*^*2*^	0.47				

PCL-R: Psychopathy Checklist-Revised; CAPP: Comprehensive Assessment of Psychopathic Personality

**Table 7:** Ridge regression predicting each higher-level dimension of both instruments (PCL-R, Cooke and Michie model).

CAPP	B	P	PCL-R	B	P
**Attachment**			**Factor 1**		
**Factor 2**	1.45	p≤ 0.001	**Cognitive**	-0.07	0.02
			**Dominance**	0.12	p≤ 0.001
			**Emotional**	-0.14	p≤ 0.001
			**Self**	0.11	p≤ 0.001
*R*^*2*^	0.49		*R*^*2*^	0.50	
**Behavioural**			**Factor 2**		
**Factor 2**	0.47	0.02	**Attachment**	0.19	p≤ 0.001
**Factor 3**	1.33	p≤ 0.001	**Dominance**	0.06	0.005
*R*^*2*^	0.38		*R*^*2*^	0.53	
**Cognitive**			**Factor 3**		
**Factor 2**	0.82	p≤ 0.001	**Behavioural**	0.19	p≤ 0.001
**Factor 3**	0.57	p≤ 0.001	**Emotional**	0.11	0.03
*R*^*2*^	0.33		*R*^*2*^	0.37	
**Dominance**			**Testlet 1**		
**Factor 1**	1.41	p≤ 0.001	**Dominance**	0.04	p≤ 0.001
**Factor 2**	2.22	p≤ 0.001	**Emotional**	-0.06	0.003
**Factor 3**	0.42	0.04	**Self**	0.09	p≤ 0.001
*R*^*2*^	0.53		*R*^*2*^	0.42	
**Emotional**			**Testlet 2**		
**Factor 2**	1.30	p≤ 0.001	**Cognitive**	-0.04	0.04
**Factor 3**	0.42	p≤ 0.001	**Dominance**	0.08	p≤ 0.001
			**Emotional**	-0.06	0.002
*R*^*2*^	0.42		*R*^*2*^	0.39	
**Self**			**Testlet 3**		
**Factor 1**	1.53	p≤ 0.001	**Attachment**	0.15	p≤ 0.001
**Factor 2**	0.93	p≤ 0.001	**Cognitive**	-0.07	0.003
			**Dominance**	0.04	0.003
*R*^*2*^	0.45		*R*^*2*^	0.55	
**Attachment**			**Testlet 4**		
**Testlet 3**	1.94	p≤ 0.001	**Attachment**	0.03	0.03
**Teslet 4**	0.74	0.001	**Dominance**	0.01	0.03
*R*^*2*^	0.52		*R*^*2*^	0.26	
**Behavioural**			**Testlet 5**		
**Teslet 5**	1.12	p≤ 0.001	**Behavioural**	0.11	p≤ 0.001
**Teslet 6**	1.49	p≤ 0.001	**Emotional**	0.06	0.03
*R*^*2*^	0.38		*R*^*2*^	0.25	
**Cognitive**			**Testlet 6**		
**Teslet 3**	0.67	p≤ 0.001	**Behavioural**	0.07	p≤ 0.001
**Teslet 4**	0.85	p≤ 0.001	**Cognitive**	0.05	0.03
**Teslet 6**	1.06	p≤ 0.001			
*R*^*2*^	0.36		*R*^*2*^	0.34	
**Dominance**					
**Teslet 1**	1.16	0.007			
**Teslet 2**	1.58	0.001			
**Teslet 3**	2.69	p≤ 0.001			
**Teslet 4**	1.41	0.004			
**Teslet 6**	0.81	0.04			
*R*^*2*^	0.54				
**Emotional**					
**Teslet 3**	1.61	p≤ 0.001			
**Teslet 4**	0.67	0.001			
**Teslet 6**	0.65	0.001			
*R*^*2*^	0.48				
**Self**					
**Teslet 1**	2.23	p≤ 0.001			
**Teslet 3**	0.93	0.003			
**Teslet 4**	1.02	0.007			
*R*^*2*^	0.47				

PCL-R: Psychopathy Checklist-Revised; CAPP: Comprehensive Assessment of Psychopathic Personality

That analysis showed the following clear associations for the Hare model: Attachment and Factor 1 & Facet 2; Behavioural and Factor 2 & Facet 3 & Facet 4; Dominance and Factor 1 & Facet 1 & Facet 2; Emotional and Factor 2; and Self and Factor 1 & Facet 1; and the following for the Cooke and Michie model: Attachment and Factor 2 & Teslet 3; Dominance and Factor 1 & Factor 2 & Teslet 3;and Self and Factor 1 & Teslet 1. The following CAPP domains were not predicted with an R2 above 0.40 (Tables 6 and 7): Cognitive with Hare´s factors & facets, and Behavioural and Cognitive with Cooke and Michie´s factors and testlets. The following PCL-R factors, facets and testlets were not predicted with an R2 above 0.40 (Tables 6 and 7): Hare´s Facet 3 and Facet 4, and Cooke and Michie´s Factor 3, Teslet 2, Testlet 4, Testlet 5, and Testlet 6. These results are clearly close to the stepwise method ones.

It is also of interest to indicate which CAPP and PCL-R symptoms showed the strongest correlations (≥0.4). These data can be found in Table 8.

**Table 8:** Strongest correlations (≥0.40) between PCL-R and CAPP symptoms.

PCL-R symptoms	Correlations with CAPP symptoms
Item 1 Glibness / superficial charm	Garrulous (CAPP dominance 30) (0.66)
	Self-aggrandising (CAPP self 14) (0.45)
	Sense of uniqueness (CAPP self 1) (0.45)
Item 2 Grandiose sense of self worth	Intolerant (CAPP cognitive 7) (0.47)
	Inflexible (CAPP cognitive 27 (0.40)
	Antagonistic (CAPP dominance 11) (0.41)
	Domineering (CAPP dominance 12) (0.53)
	Garrulous (CAPP dominance 30) (0.50)
	Self-centred (CAPP self 20) (0.50)
	Self-aggrandising (CAPP self 14) (0.75)
	Sense of uniqueness (CAPP self 1) (0.67)
	Sense of entitlement (CAPP self 13) (0.51)
	Sense of invulnerability (CAPP self 22) (0.53)
Item 3 Need for stimulation/proneness to boredom	Uncommitted (CAPP attachment 8) (0.43)
	Reckless (CAPP behavioural 15) (0.42)
	Lacks planfulness (CAPP cognitive 29) (0.40)
Item 4 Pathological lying	Deceitful (CAPP dominance 10) (0.48)
	Manipulative (CAPP dominance 9) (0.51)
	Insincere (CAPP dominance 23) (0.46)
	Garrulous (CAPP dominance 30) (0.48)
Item 5 Conning/manipulative	Uncaring (CAPP attachment 24) (0.45)
	Deceitful (CAPP dominance 10) (0.44)
	Manipulative (CAPP dominance 9) (0.55)
	Insincere (CAPP dominance 23) (0.57)
	Lacks anxiety (CAPP emotional 5) (0.40)
	Lacks remorse (CAPP emotional 16) (0.45)
	Self–centred (CAPP self 20) (0.47)
	Sense of entitlement (CAPP self 23) (0.41)
Item 6 Lack of remorse or guilt	Detached (CAPP attachment 18) (0.42)
	Uncommitted (CAPP attachment 8) (0.45)
	Unempathic (CAPP attachment 25) (0.40)
	Uncaring (CAPP attachment 24) (0.41)
	Manipulative (CAPP dominance 9) (0.41)
	Lacks remorse (CAPP emotional 16) (0.55)
	Self-justifying (CAPP self 2) (0.42)
Item 7 Shallow affect	Detached (CAPP attachment 18) (0.47)
	Uncommitted (CAPP attachment 8) (0.45)
	Aggressive (CAPP behavioural 32) (0.43)
	Intolerant (CAPP cognitive 7) (0.42)
	Inflexible (CAPP cognitive 27 (0.40)
	Antagonistic (CAPP dominance 11) (0.45)
	Domineering (CAPP dominance 12) (0.50)
	Lacks anxiety (CAPP emotional 5) (0.44)
	Lacks emotional depth (CAPP emotional 4) (0.58)
	Lacks remorse (CAPP emotional 16) (0.45)
	Self–centred (CAPP self 20) (0.40)
Item 8 Callous/lack of empathy	Detached (CAPP attachment 8) (0.54)
	Uncommitted (CAPP attachment 8) (0.53)
	Unempathic (CAPP attachment 25) (0.77)
	Uncaring (CAPP attachment 24) (0.59)
	Aggressive (CAPP behavioural 32) (0.52)
	Suspicious (CAPP cognitive 19) (0.45)
	Intolerant (CAPP cognitive 7) (0.51)
	Inflexible (CAPP cognitive 27) (0.44)
	Antagonistic (CAPP dominance 11) (0.49)
	Domineering (CAPP dominance 12) (0.59)
	Deceitful (CAPP dominance 10) (0.42)
	Manipulative (CAPP dominance 9) (0.46)
	Lacks anxiety (CAPP emotional 5) (0.46)
	Lacks emotional depth (CAPP emotional 4) (0.52)
	Lacks remorse (CAPP emotional 16) (0.51)
	Self-centred (CAPP self 20) (0.49)
	Self-aggrandising (CAPP self 14) (0.49)
	Sense of uniqueness (CAPP self 1) (0.42)
	Self-justifying (CAPP self 2) (0.41)
Item 9 Parasitic lifestyle	Lacks perseverance (CAPP behavioural 3) (0.48)
	Unreliable (CAPP behavioural 26) (0.40)
	Reckless (CAPP behavioural 15) (0.41)
	Disruptive (CAPP behavioural 17) (0.44)
	Lacks concentration (CAPP cognitive 28) (0.45)
	Lacks planfulness (CAPP cognitive 29) (0.47)
	Lacks anxiety (CAPP emotional 5) (0.42)
	Lacks emotional stability (CAPP emotional 31) (0.41)
Item 10 Poor behavioural controls	
Item 11 Promiscuous sexual behaviour	
Item 12 Early behavioural problems	Uncaring (CAPP attachment 24) (0.42)
	Lacks Perseverance (CAPP behavioural 3) (0.42)
	Reckless (CAPP behavioural 15) (0.43)
	Restless (CAPP behavioural 6) (0.41)
	Disruptive (CAPP behavioural 17) (0.46)
	Lacks concentration (CAPP cognitive 28) (0.40)
	Lacks planfulness (CAPP cognitive 29) (0.43)
	Lacks emotional stability (CAPP emotional 31) (0.40)
Item 13 Lack of realistic, long-term goals	Uncaring (CAPP attachment 24) (0.40)
	Lacks perseverance (CAPP behavioural 3) (0.53)
	Unreliable (CAPP behavioural 26) (0.44)
	Reckless (CAPP behavioural 15) (0.42)
	Disruptive (CAPP behavioural 17) (0.43)
	Lacks planfulness (CAPP cognitive 29) (0.48)
Item 14 Impulsivity	
Item 15 Irresponsibility	
Item 16 Failure to accept responsibility for own actions	Lacks remorse (CAPP emotional 16) (0.44)
Item 17 Many short-term marital relationships	
Item 18 Juvenile delinquency	
Item 19 Revocation of conditional release	
Item 20 Criminal versatility	

PCL-R: Psychopathy Checklist-Revised; CAPP: Comprehensive Assessment of Psychopathic Personality

CAPP correlations between Total and Domain scores for the whole sample, in inmates with a PCL-R of 25 or more, and inmates with a PCL-R of 30 or more can be found in Table 9.

**Table 9:** As previously reported in [37], Table 7 indicates correlations among CAPP (total and domains scores) for the whole sample, for inmates with a PCL-R cut-off score of 25 or superior (italics), and for inmates with a PCL-R cut-off score of 30 or superior (underlined).

	Attachment	Behavioural	Cognitive	Dominance	Emotional	Self	Total
Attachment	**1.00**						
Behavioural	0.55**	**1.00**					
	*0*.25*						
	0.50*						
Cognitive	0.70**	0.74**	**1.00**				
	*0*.56**	*0*.57**					
	0.68**	0.67**					
Dominance	0.79**	0.62**	0.77**	**1.00**			
	*0*.68**	*0*.40**	*0*.62**				
	0.66**	0.74**	0.79**				
Emotional	0.79**	0.60**	0.77**	0.91**	**1,00**		
	*0*.71**	*0*.37*	*0*.64**	*0*.83**			
	0.70**	0.55*	0.83**	0.88**			
Self	0.67**	0.59**	0.66**	0.83**	0.73**	**1,00**	
	*0*.45**	*0*.36*	*0*.35*	*0*.70**	*0*.45**		
	0.49*	0.59*	0.54*	0.77**	0.56*		
Total	0.84**	0.78**	0.87**	0.95**	0.91**	0.87**	**1,00**
	*0*.75**	*0*.63**	*0*.77**	*0*.92**	*0*.83**	*0*.74**	
	0.75**	0.82**	0.85**	0.95**	0.86**	0.80**	

CAPP: Comprehensive Assessment of Psychopathic Personality;

* 0.05 < p ≥ 0.001;

** p < 0,001

## Discussion

The purpose of this study was to compare PCL-R and CAPP scores in a large non-convenience sample following standard assessment. To our knowledge, this is the first study where this analysis has been conducted.

### Intercorrelations between the PCL-R and the CAPP

As in previous research [36], in our whole sample, PCL-R total score and CAPP total score showed an strong association as seen in the linear regression models and in the correlation study (Tables 2 & 3). In the whole sample, the PCL-R life assessment, both models, and the CAPP six-month evaluation displayed a strong and congruent association. For the whole sample, we found strong and congruent associations between the PCL-R and the CAPP not only for total scores but also for domains and factors, even when considering different PCL-R models. As in previous research, the CAPP total score and most of its domains Attachment, Emotional, Dominance and Self, showed the strongest association with PCL-R affective and interpersonal scores (Factor 1 in the Hare model, and factors 1 and 2 in the Cooke and Michie model), but, as expected, only the Behavioural domain had a strong association with PCL-R social deviance scores (Factor 2 in the Hare model, and factor 3 in the Cooke and Michie model) (Sandvik et al., 2012). The same relational pattern was found when regressions with the stepwise and Ridge methods were used (Tables 4, 5, 6 & 7), although these methods allow a deeper analysis. Attachment is clearly more associated with being callous and unemotional (Factor 1 & Facet 2 in the Hare model; factor 2 & testlets 3 and 4 (*items 6 and 16*) in the Cooke and Michie model), while Emotional is also connected with being callous and unemotional (Facet 2 in the Hare model; factor 2 & testlet 3 in the Cooke and Michie model) but also with changes in lifestyle (Factor 2 & facet 4 in the Hare model; factor 3 & testlet 6 in the Cooke and Michie model). Dominance is clearly associated with being both callous and unemotional as well as narcissistic (Factor 1 & facets 1 and 2 in the Hare model; factors 1 and 2 & testlets 1,2 (*items 4 and 5*),3 and 4 in the Cooke and Michie model). So, these strong associations show that Dominance measures behaviour related to psychopathic personality traits [10, 11]. This makes Dominance one of the most central and prototypical CAPP domains [31, 33, 34], and assessing its items is the best way to start evaluating how callous, unemotional and narcissistic an inmate is. The Self domain retains a strong association with being narcissistic (Factor 1 & facet 1 in the Hare model; factor 1 & testlet 1 in the Cooke and Michie model). The same applies to the Behavioural domain which maintains its strong associations with conduct disorder (Factor2 & facets 3 and 4 in the Hare model; factor 3 & testlets 5 (*items 2*, *14 and 15*) and 6 in the Cooke and Michie model). The Cognitive domain, one of the least prototypical CAPP domains in previous research [31, 33], showed the weakest associations in the stepwise and Ridge regressions methods. This points to the fact that this domain is less central to the CAPP psychopathy concept.

When a cut-off score of 25 was introduced, resulting in a sample with more psychopathic inmates, we found a general drop in correlations between the PCL-R and the CAPP, although we still found congruent correlations for total values and also for domains and factors.

How is this possible? Should it not be logical to think that, as inmates become more psychopathic, they become a more homogeneous group, and because of this, that correlations become stronger and more significant? Or is it just that, as we reduce the range of values that we are correlating, the lower those correlations will be.

The case of the sub-sample with cut-off scores of 30 or more can help elucidate these contradictory findings. In this group there is a general increase in the strength and significance of most of the correlations, although not as strong as in the whole sample. This is unexpected based solely on the range restriction effect. Then, for inmates above the cut-off score of 30 we found a mild general increase in correlations between the PCL-R and the CAPP, mainly in total values, with many changes in correlation strength and significance that point to a better fit among all groups between items in both instruments that assess deficient affective experience and pervasive impulsive behaviour.

To summarize, in the whole sample, correlations are high between lifetime PCL-R and present-time CAPP. There seems to be concurrence regarding out who is highly psychopathic and who is not psychopathic at all. But in between we find a grey area of lower correlations that goes beyond the expected range restriction effect. Bearing in mind that correlations above PCL-R cut-off scores of 30 are not as large as in the whole sample, but higher than those above the PCL-R cut-off scores of 25, analysis of this grey area provides relevant information. It suggests that PPD is more dimensional than categorical [12, 14, 16, 22]. When values are high enough for a clear PPD diagnosis, the association of an affective dimension versus a behavioural one becomes clearer, but without a unique or categorical pattern of correlation. This dimensional pattern can be found for correlations between the PCL-R and CAPP values when considering the whole sample or just inmates above the cut-offs. Better correlations in all analyses between Attachment and Emotional domains with facet 2 and testlet 3 compared with facet 1 and testlets 1 and 2 indicate that affective and interpersonal are also independent dimensions. Knowing that an inmate scores above one of the cut-offs is not the important issue. What is most important is establishing what dimension contributes the most to this high increase in the total score.

### Criminal behaviour as a defining trait of PPD

When considering only PCL-R scores and models, just as in previous research, the picture is not so clear [5, 10, 11, 20, 24, 25, 42, 43]. Facet 4, which comprises all the criminal items, shows correlation values with CAPP domains and CAPP total score that, in general, resemble those of the other facets and testlets (Tables 2 and 3). So, for the PCL-R, removal of those items does not seem to improve its correlation values, as we can see when we compare the correlation values of Hare´s Factor 2 (Facets 3 and 4) versus the correlations values of Cooke and Michie´s Factor 3 (Testlets 5 and 6 equivalent to Hare´s facet 3) (Tables 2 and 3). PCL-R criminal scores can be analysed from another perspective. Facet 4 is needed to score 30 or higher on the PCL-R, and in this group of inmates, Facet 4 has a correlation close to 1 (0.97) with factor 2. This, tells us that to surpass this cut-off score, levels of criminality must increase, and so they do as PCL-R scores rise. As this happens, its correlation with PCL-R total and CAPP total scores and domain scores improves when compared with other PCL-R facets and testlets, even when there is range restriction, it is actually the only facet or testlet that shows such a general increase in its correlation strength and significance between the two PCL-R cut-offs. So, showing criminal behaviour does not mean that someone is psychopathic, but that inmates who are more psychopathic, from a PCL-R perspective do have high criminality scores. And these high criminality scores are needed to surpass the cut-off score of 30. In conclusion, in this sample when using the PCL-R cut-off score of 30, an inmate cannot have a PPD diagnosis if he or she does not have a facet 4 score close to 100%. Then, we can establish that, in the current sample, inmates above the cut-toff score of 30 have homogeneously high criminal (Facet 4) scores, and if we use this cut-off for the diagnosis of PPD, we arrive at the artificial conclusion, that criminality is strongly related to PPD, when it is just only a matter of scoring and setting a cut-off. We do not find such a strong relationship when we use the cut-off score of 25.

It should also be borne in mind that the IPDE antisocial diagnosis, a mix of affective-interpersonal-behavioural symptoms, is significantly associated with PCL-R and CAPP scores, at the univariate level (Table 1) for both instruments, and at the multivariate (regression analysis) level only with the CAPP, while type (drug dealing and property crimes more than violent crimes) and number of official charges directly associated with criminality scores are more significantly associated with PCL-R scores, as expected. Driving while intoxicated, mainly under the influence of alcohol, has an inverse association with PCL-R but not CAPP scores. This is because, under Spanish law, recidivist drunk drivers, who normally do not display more criminal behaviour, are generally and more easily sent to prison. Drug use is significantly more associated with PCL-R than CAPP scores because inmates have to fund their drug use through crime, particularly drug dealing and property crimes and these increase factor 2 scores, a well-known interaction [44, 45]. Accordingly, for the cheapest substance, alcohol, we find the opposite association.

The CAPP, without criminality items showed better correlation of its domains when compared with the PCL-R models (Tables 2, 3 and 9). Some of its domains even correlated better with PCL-R facets and testlets than the facets and testlets did among themselves (Tables 2 and 3). Dominance, which shows a clear strong correlation with emotional, is the dimension that has the strongest correlations with all the other CAPP dimensions, once again showing its central position in the CAPP psychopathy concept. From this perspective, less psychopathic inmates do not show dominant behaviour while more psychopathic ones do show intense dominant behaviour. Table 1 and the regression analysis indicate that CAPP is more associated with IPDE personality disorders than with substance use and official charges, pointing, as expected, to a more intense correlation with general psychopathology than with antisocial behaviour, as was intended by the developers of the CAPP model [29].

### Limitations

This study has some limitations that should be considered. Although the researchers meticulously followed the blind procedure, described elsewhere [37, 38], it may have been broken by inmates when interviewed. This is an inescapable limitation, as they have to be interviewed with a semi-structured approach, which allows the blinding to be broken. PCL-R, CAPP and IPDE evaluations were conducted by just one researcher; this circumstance enhances the study´s internal validity, but may reduce its external validity. The contrast in IPDE Personality Disorders prevalence in the present study in comparison with former research may be a sign of this bias [46], but the prevalence of Personality Disorder varies extensively among studies [46] and some have reported rates similar to the ones found in the current study [47]. PCL-R scores in the present study are similar to those of other international studies, but with lower antisocial scores [25]. These lower facet 4 scores are easily explainable as our non-convenience sample is from a low-medium security institution that houses less highly antisocial inmates [25]. Nevertheless, due to the provenance of their samples, former UK studies arrived at even lower scores, and in the current study, as in former ones, higher scores for factor 2 were also found when compared with factor 1 [25].

We also have to take into account that including the PCL-R factors in the correlation study (Tables 2 and 3) may increase the risk of Type I errors.

## Conclusions

A high correlation was found between PCL-R and CAPP scores in the whole sample, but as scores increased, the correlations weakened. In inmates above the PCL-R cut-off score of 30, correlations again improved. All these data indicate that PPD, understood to mean having high scores on the PCL-R and CAPP, is a multidimensional entity, and inmates can develop the disorder, and then receive the diagnosis through different dimensions. This complex relationship should be borne in mind by clinical and forensic personnel involved in the assessment of PPD for legal or treatment purposes. The CAPP domains showed better correlations among all sub-samples when compared with the PCL-R factors, and this finding shows that a tool for the assessment of psychopathy without criminality items is valuable for clinical assessment and research purposes. Through all the analyses, the CAPP showed a strong association with the PCL-R (total score, items and models). This association indicates that the CAPP, without criminality items, assesses the same underlying psychopathy construct as the PCL-R.

## Supporting information

S1 Database (XLS)
